# Role of matrix metalloproteinases in the pathogenesis of idiopathic pulmonary fibrosis

**DOI:** 10.1186/s12931-016-0343-6

**Published:** 2016-03-04

**Authors:** Annie Pardo, Sandra Cabrera, Mariel Maldonado, Moisés Selman

**Affiliations:** Facultad de Ciencias, Universidad Nacional Autónoma de México, México, DF Mexico; Instituto Nacional de Enfermedades Respiratorias Ismael Cosío Villegas, México, DF Mexico

**Keywords:** Lung fibrosis, IPF, Metalloproteinases, Matrisome, MMP

## Abstract

Idiopathic pulmonary fibrosis (IPF) is a progressive and devastating lung disorder of unknown origin, with very poor prognosis and no effective treatment. The disease is characterized by abnormal activation of alveolar epithelial cells, which secrete numerous mediators involved in the expansion of the fibroblast population, its differentiation to myofibroblasts, and in the exaggerated accumulation of extracellular matrix provoking the loss of lung architecture. Among the excessively produced mediators are several matrix metalloproteases (MMPs) which may contribute to modify the lung microenvironment by various mechanisms. Thus, these enzymes can not only degrade all the components of the extracellular matrix, but they are also able to release, cleave and activate a wide range of growth factors, cytokines, chemokines and cell surface receptors affecting numerous cell functions including adhesion, proliferation, differentiation, recruiting and transmigration, and apoptosis. Therefore, dysregulated expression of MMPs may have profound impact on the biopathological mechanisms implicated in the development of IPF. This review focuses on the current and emerging evidence regarding the role of MMPs on the fibrotic processes in IPF as well as in mouse models of lung fibrosis.

## Background

### Idiopathic pulmonary fibrosis

Idiopathic pulmonary fibrosis (IPF), the most aggressive fibrotic lung disorder, is a chronic, progressive, irreversible, and usually lethal lung disease of unknown etiology [1, 2].

IPF is an aging-associated disease usually found in people over 50 and its incidence and prevalence increases markedly in the elderly. Although the underlying mechanisms linking aging with IPF are not fully understood, it has been hypothesized that IPF patients may have an accelerated process of lung aging, characterized by increased genomic instability, abnormal shortening of telomeres, epithelial cell senescence, mitochondrial dysfunction, and loss of proteostasis, among others [3, 4]. However, how these mechanisms of aging interrelate is currently largely unknown. In this context, it has been proposed that IPF is the consequence of the convergence of three conditions, a genetic architecture that results in an easy loss of the alveolar epithelial integrity, “accelerated” aging, and a distinctive epigenetic “profibrotic” modification [4, 5]. This “mechanistic convergence model” hypothesizes that IPF is driven by the co-incidence of multifactorial components which results in a distinct pathogenic cascade leading to the aberrant activation of alveolar epithelial cells [1, 4, 6]. This notion is supported by the presence of increasing numbers of phenotypically varied epithelial cells which are exceptionally active, and synthesize nearly all the mediators that contribute to the formation and activation of the fibroblastic foci and to the progressive and exaggerated accumulation of extracellular matrix. Among these, various MMPs have been found dysregulated and evidence indicates that play a central role not only in the abnormal tissue remodeling but also influencing the epithelial and mesenchymal cell behavior [7] Table 1.

**Table 1:** Matrix metalloproteinases in pulmonary fibrosis

MMP	IPF	Murine fibrosis model	In vitro related studies	References
MMP1 Collagenase 1	↑Plasma ↑Serum ↑ BAL ↑ Lung Expressed by AECs and AM	No data in Mmp1a mouse (McolA)	Bidirectional correlation with HIF1α in AECs, represses mitochondrial oxygen consumption	[7, 19, 32, 47]
MMP2 Gelatinase A	↑ BAL ↑Lung Expressed by BECs, AECs, fibroblasts and fibrocytes.	Not done in Mmp2 null mice	-	[27, 46, 63]
MMP3 Stromelysin 1	↑ Serum ↑ BAL ↑Lung Expressed by BECs, AECs, AM, and fibroblasts	↑ Lung Mmp3-null mice protected from bleo fibrosis.	Induces epithelial-mesenchymal transition	[35–37, 48, 70]
MMP7 Matrilysin	↑Plasma ↑Serum ↑BAL ↑Lung Expressed by BECs and AECs	Mmp7-null mice protected from bleo fibrosis	Shows bidirectional correlation with osteopontin	[22, 23, 47]
MMP8 Collagenase 2	↑Plasma ↑BAL ↑Lung Expressed by blood monocytes, AM, BECs, AECs and fibrocytes	↑ Lung Mmp8-null mice protected from bleo fibrosis	Facilitates fibrocytes migration	[37, 46, 48, 50–52]
MMP9 Gelatinase B	↑BAL ↑Lung Expressed by AECs, neutrophils, AM, fibrocytes, and fibroblasts	↑ Lung Mmp9-null mice no change with WT in bleo fibrosis MMP9-over-expression in AM reduces fibrosis	Expressed by Thy-1 (−) lung fibroblasts with TGF-β1; enhances fibroblasts migration	[7, 39, 40, 42, 46, 48]
MMP10 Stromelysin 2	↑ Serum ↑ BAL Expressed by AM, BECs and AECs	↑ Lung Not done in Mmp10-null mice	-	[60, 71]
MMP11 Stromelysin 3	^a^	Not done in Mmp11-null mice	-	
MMP12 Macrophage elastase	↑ BAL	↑ Lung Mmp12-null mice no changes with WT in bleo fibrosis	-	[37, 57–59]
MMP13 Collagenase 3	↑ Lung Expressed by AM, BECs and AECs	↑ Lung Mmp13-null mice increased bleo fibrosis protected from radiation fibrosis	-	[54–56]
MMP14 MT1-MMP	↑ Lung Expressed by AECs, AM and endothelial cells.	↑ Lung Deficient mice die between 20 and 90 days after birth	-	[27, 36, 37, 60, 62, 63, 74]
MMP15 MT2-MMP	↑ Lung Expressed by endothelial and AECs	No data.	-	[63]
MMP16 MT3-MMP	Expressed by fibroblasts and AECs	No data	Upregulated in fibroblasts by TGF-β1	[63]
MMP17 MT4-MMP	^a^	No data	-	
MMP19	↑ Upregulated in hyperplastic AECs	↑ Lung Mmp19-null mice increased bleo fibrosis	Positive correlation with COX2 in AECs. Induce an antifibrotic phenotype in fibroblasts	[24–26, 37]
MMP20 Enamelysin	^a^	Not done in Mmp20-null mice	-	
MMP21	^a^	No data.	-	
MMP23B	^a^	Not done in Mmp23- null mice	-	
MMP24 MT5-MMP	Expressed by basal BECs, and in areas of squamous metaplasia	Not done in Mmp24-null mice	-	[63]
MMP25 MT6-MMP	↓ Lung Cell type localization unknown.	No data	-	[7]
MMP26 Matrilysin-2	^a^	Absent in mouse	-	
MMP27	^a^	No data.	-	
MMP28 Epilysin	↑Lung	Mmp28-null mice reduced bleo fibrosis	Protects BECs and AECs from apoptosis	[7, 64, 75]

^a^ the expression of these MMPs has not been found altered in IPF vs normal lungs by microarrays

*BECs* bronchiolar epithelial cells

*AM* alveolar macrophages

*BAL* bronchiolar lavage

*AECs* alveolar epithelial cells

*Bleo* bleomycin-induced

### Matrix metalloproteases

MMPs, the zinc-dependent matrixins belong to the M10A subfamily of metallopeptidases, and have long been considered as the principal effectors of extracellular matrix (ECM)/core matrisome proteins degradation. The contribution of MMPs to the matrisome remodeling is vastly complex since these endopeptidases have a broad scope of influence. They are not only responsible for ECM degradation, but also shed cell membrane proteins, and process and cleave diverse bioactive mediators such as growth factors, cytokines and chemokines, considered as matrix associated proteins, modulating their activity either by direct cleavage, or releasing them from extracellular matrix bound stocks [8, 9].

In humans the MMP family consist of 24 genes including two genes for MMP23 and in mice, in which the experimental models of pulmonary fibrosis are mainly performed, MMPs comprise 23 genes. In mice, human MMP1 gene (collagenase 1) is represented by Mmp1a and Mmp1b genes, they have only one Mmp23 gene, and lack MMP26 (matrilysin-2) gene [10].

Numerous studies have shown that MMPs are strongly regulated at multiple levels, starting from transcriptional regulation of gene expression through growth factors, cytokines, hormones, and cell-extracellular matrix, and cell-cell interactions. Post-transcriptional regulatory processes include mRNA stability, protein translational efficiency, and regulation by microRNAs. After translation, zymogen activation of different MMPs may take place intracellularly, at the cell surface, or in the extracellular space. Then, the activity of MMPs is inhibited by different mechanisms and molecules, such as the TIMP family of proteins (TIMPs), α2-macroglobulin, and the membrane-associated RECK (reversion-inducing Cys-rich protein with Kazal motifs) [11, 12].

Most of the MMPs are secreted enzymes, although there are also membrane type MMPs (MT-MMPs), and even some MMPs have been reported in intracellular organelles, including nuclear localization acting on intracellular substrates, or as transcription factors [13–18].

Under normal conditions, their activity is low but increases during repair or remodeling processes and in several pathological conditions. Traditionally, as MMPs have been considered mainly as proteases involved in the degradation of ECM proteins, it has been hypothesized that pathologic fibrotic scars represent an improper balance between deposition (excessive) and degradation (deficient) of ECM components.

However, the finding that some MMPs are highly expressed in a complex fibrotic disease such as IPF seemed to contradict this postulate and numerous evidence suggest that this simplistic concept is not only insufficient but furthermore, probably wrong.

**Fig. 1:**
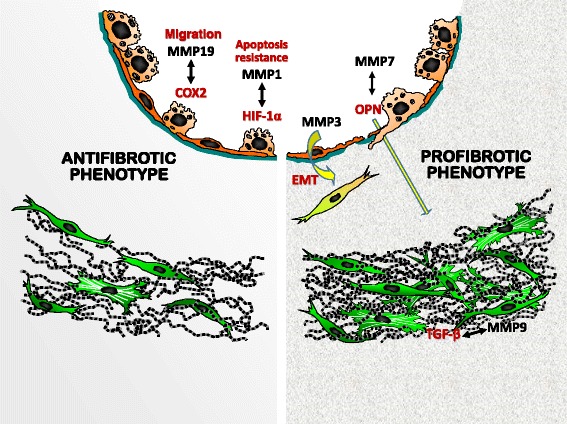
Putative roles of some MMPs in pulmonary fibrosis. In activated alveolar epithelial cells, MMP-7 cleaves osteopontin and potentiates its function which in turn upregulates and activates MMP-7. In this profibrotic cross-talk, osteopontin upregulates the expression of ECM proteins. MMP-19 co-localizes and co-regulates with COX2 which end product, prostaglandin E2, is a potent suppressor of fibroblast proliferation and collagen production. Deficiency of MMP-19 in fibroblasts results in the upregulation of several profibrotic genes and pathways that provoke an increase of fibroblast migration and proliferation and a decrease of these processes in epithelial cells. Upregulation of MMP-1 in alveolar epithelial cells represses mitochondrial respiration and oxidative stress, while promotes cell proliferation, migration, and HIF-1α expression, and induces an anti-apoptotic phenotype. MMP3 induces EMT, and MMP9 is upregulated by TGF β in Thy-1 negative fibroblasts which in turn activates this growth factor. OPN = osteopontin; COX2 = cyclooxygenase 2; ECM = extracellular matrix; EMT = epithelial to mesenchymal transition

In this review, key pathophysiological roles for MMPs in IPF will be discussed herein paying special attention to those that have been shown to be upregulated, which have known correlations with profibrotic or anti-fibrotic mediators, and have mechanistic information through experimental models or in vitro studies to understand their participation in the pathogenesis of the disease (Fig. 1).

#### MMP7 a profibrotic mediator

MMP7 is one of the molecules most highly expressed in IPF when compared with normal lungs or lung tissues from other interstitial lung diseases and is localized primarily in the aberrantly activated alveolar epithelial cells (AECs) and in bronchiolar epithelial cells [19, 20].

It has been postulated that MMP7 plays a profibrotic role based on the finding that MMP7 deficient mice are protected from bleomycin induced lung fibrosis. Its profibrotic role might be multiple considering its broad substrate specificity that includes basement membrane and ECM components. Additionally, MMP7 processes numerous bioactive substrates including FAS ligand (FasL), β4 integrin, E-cadherin, plasminogen, transmembrane tumor necrosis factor α (pro-TNF-α), syndecan, insulin growth factor binding protein-3 (IGFBP-3) among others [21]. Interestingly, in the AECs of IPF lungs MMP7 co-localizes with osteopontin a multifunctional cytokine involved in cell adhesion and migration suggesting that this interaction may have an important effect on IPF [22]. This hypothesis is supported by the finding that MMP7 is induced and activated by osteopontin while the latter is cleaved and activated by MMP7 [23]. Additionally, osteopontin increases the expression of type 1 collagen and TIMP1 and decreases MMP1 on lung fibroblasts supporting the notion that this bidirectional regulation between MMP7 and osteopontin plays a profibrotic role in IPF.

#### MMP19 an antifibrotic mediator

The evaluation of the transcriptional signature of hyperplastic epithelial cells compared with conserved epithelial cells in the same lungs revealed that MMP19 is highly upregulated in hyperplastic epithelial cells adjacent to fibrotic regions [24]. Interestingly, MMP19 overexpression displayed a positive correlation with prostaglandin-endoperoxide synthase 2 (PTGS2; COX2), which was also upregulated in hyperplastic alveolar epithelial cells and co-localized with MMP19 [24].

Surprisingly, Mmp19 deficient mice displayed an exaggerated lung fibrotic response to bleomycin compared with WT mice suggesting that MMP19 plays a protective role [24]. Interestingly, COX-2 has been also suggested as protective against pulmonary fibrogenesis [25]. Therefore, it can be hypothesized that the increased expression of MMP19 and COX-2 in the hyperplastic epithelial cells represents a normal but potentially insufficient response to continuous epithelial injury/activation in IPF lungs.

Supporting an antifibrotic role of MMP19, lung fibroblasts lacking this enzyme exhibit dysregulation of several profibrotic pathways and show a substantial increase in proliferation and transmigration [26]. These results suggest that, in lung fibroblasts, MMP19 has strong regulatory effects on the synthesis of key ECM components, supporting the finding of a stronger fibrotic response observed in the lungs from MMP19 deficient mice.

#### The paradox of MMP1 overexpression in IPF

Transcriptional and immunohistochemical analyses have consistently revealed that MMP1 is significantly overexpressed in IPF lungs compared with controls [7, 27].

This represented an intriguingly finding since MMP1 is an enzyme capable of cleaving fibrillar collagens, the typical excessively accumulated ECM molecules in IPF. Furthermore, MMP1 is associated with pathologies characterized by excessive ECM degradation, such as rheumatoid arthritis and lung emphysema [28–31].

A partial explanation for this paradox is that in IPF lungs, MMP1 is localized primarily in the reactive alveolar epithelium while is virtually absent in fibroblasts in the interstitial compartment where collagens are being accumulated.

Transfection of MMP1 in alveolar epithelial cells indicates that this enzyme induces cell proliferation, accelerates wound closing and protects cells from apoptosis [32]. Importantly, MMP1 was identified in the mitochondria of AECs and repressed mitochondrial respiration, induced the expression of hypoxia-inducible factor-1α (HIF-1α) under normoxic conditions, and decreased the production of both mitochondrial and total reactive oxygen species (ROS) [32]. Moreover, MMP1 was also up regulated when HIF-1α was induced by hypoxia in AECs suggesting a bidirectional cross talk. Altogether, these data suggest a complex role of MMP1 in IPF, which goes far beyond of digesting fibrillar collagens.

Mmpla, the putative murine orthologue of MMP1 shows low identity (58 % in amino acids) [33], and does not seem to play a similar role in mice as MMP1 in humans [34]. It is expressed less ubiquitously in healthy mouse tissue than in humans and their expression seems to be restricted exclusively to reproductive tissues since it was found only in the placenta and in the testes, while in humans plays a more fundamental role in tissue remodeling processes. Importantly, it is absent in the embryo, lung, kidney, brain, heart, muscle, and liver [33, 34]. In this context, the classical approach of using Mmp1a deficient mice as a tool to explore the participation of MMP1 in experimental pulmonary fibrosis would be far from being informative.

#### MMP3 may contribute to epithelial-mesenchymal transition

It has been found that MMP3 is upregulated in IPF lungs as well as in bleomycin induced lung fibrosis [35–37]. Moreover, Mmp3-null mice were protected from bleomycin-lung injury [35]. Furthermore, transient adenoviral vector-mediated expression of recombinant MMP3 in rat lungs resulted in accumulation of myofibroblasts and fibrosis. MMP3 is expressed by diverse cell types in IPF lungs, including bronchioalveolar epithelial cells, alveolar macrophages, and fibroblasts [35]. Interestingly, treatment of lung epithelial cells with MMP3 resulted in activation of the β-catenin signaling pathway with the subsequent induction of several β-catenin-target genes and of epithelial-mesenchymal transition [35]. These findings suggest that MMP3 may be mechanistically involved in the pathogenesis of IPF through the induction of epithelial to mesenchymal transition.

#### Expression of MMP9 is associated with the absence of Thy-1 receptor in lung fibroblasts

MMP9 is also increased in IPF lungs where it is expressed by alveolar epithelial cells, macrophages, neutrophils and fibroblasts in fibroblastic foci [27]. The finding that lung fibroblasts in this disease synthesize MMP9 is intriguing since these cells do not express this enzyme in vitro, but a finding in our laboratory opened some light to this result. Thy-1, a glycophosphatidylinositol-linked glycoprotein, is expressed by most fibroblasts from normal lungs while IPF fibroblasts are usually Thy-1 negative. Actually, loss of Thy-1 expression in fibroblasts correlates with regions of active fibrogenesis [38]. Recently, it was demonstrated that Thy-1(−) lung fibroblasts stimulated with TGF-β1 expressed MMP9, through activation of ERK1/2 signaling pathway, while Thy-1 (+) cells did not [39]. Moreover, treatment of Thy-1(−) fibroblasts with β-glycan, a TGF-β receptor antagonist, abolished MMP9 induction. These findings suggest that in the microenvironment of IPF lungs fibroblasts/myofibroblasts, which do not express Thy-1, when stimulated by the epithelial-produced TGF-β1 synthesize MMP9. Importantly, among many effects, MMP9 is also able to activate TGF-β1 contributing to enhance the pool of active TGF-β1 [40]. Therefore, TGF-β induction of MMP9 in Thy-1 (−) fibroblasts could be part of a fibrogenic feedback loop in IPF lungs.

In contrast, results in experimental models have given some puzzling results. MMP9 is usually found elevated in lung tissue homogenates and bronchoalveolar lavage fluids obtained from bleomycin-treated mice. Interestingly, the absence of this enzyme did not seem to influence the severity of fibrosis after intratracheal bleomycin [41]. However, the presence of hypertrophied and hyperplastic cuboidal epithelial cells, a usual epithelial change occurring in regions of alveolar injury, was observed in Mmp-9 (+/+) but hardly found in the Mmp-9 deficient mice. The reason of this finding is unknown but it can be speculated that MMP9 may facilitate distal airway epithelial cells to migrate into sites of alveolar injury. By contrast, transgenic overexpression of human MMP9 in macrophages attenuates lung fibrosis [42].

#### Role of MMPs in the traffic of fibrocytes to the IPF lungs

Fibrocytes are bone marrow derived cells characterized by the expression of fibroblast and leukocyte markers. They circulate in peripheral blood and appear to be a source of fibroblasts/myofibroblasts that participate in the mechanisms of wound healing and tissue fibrosis [43]. The percentage of circulating fibrocytes is significantly increased in patients with IPF, mainly in those suffering an acute exacerbation [44]. It has been proposed that the main mechanism of fibrocyte homing to the lungs is the CCL12/CXCR4 axis [45]. Lately, it was found that fibrocytes strongly express several MMPs including MMP2, MMP9, MMP8 and MMP7 [46]. Importantly, MMP8, MMP2 and MMP9 seem to participate in the process of tissue migration and homing since the movement of fibrocytes through collagen I is highly associated with the expression of collagenase MMP8 while its transmigration through basement membrane-like proteins is associated to MMP2 and MMP9 [46]. Therefore, the synthesis of these enzymes may facilitate the transendothelial and tissue migration of fibrocytes and also contribute in the remodeling of ECM during the development of IPF.

#### MMP8 may contribute to the inflammatory response in experimental lung fibrosis

Some studies have shown that MMP-8 levels are elevated in plasma, lung homogenates and BAL fluids from IPF patients [47–50]. Also, it has been reported that Mmp8 increases in the lungs of mice early after bleomycin treatment remaining elevated until 21 days [51]. In a long-term study, the expression of this enzyme was increased at the inflammatory phase, stayed up until 8 weeks post-bleomycin (the peak of fibrosis) then decreasing during fibrosis resolution [37].

In the experimental model, this enzyme may enhance inflammation and inflammatory-driven fibrosis cleaving IL10, MIP-1a and Cxcl10, and actually, Mmp8-null mice are protected from bleomycin-induced lung fibrosis although the molecular mechanisms are yet unclear [52]. Supporting a role in inflammatory disorders that evolve to fibrosis we have demonstrated that MMP8 together with MMP9 associated with neutrophils were increased in hypersensitivity pneumonitis and correlated with the development of lung fibrosis [53]. However, its role in the pathogenesis in IPF is uncertain.

#### MMP13 is deregulated but its role is uncertain

MMP13 one of the enzymes able to cleave fibrillar collagens has recently been reported to be increased at mRNA and protein level in IPF. It was mainly localized in alveolar and bronchiolar epithelial cells, and in alveolar macrophages [54]. Its role in lung fibrosis experimental models is uncertain and results obtained so far are conflicting. Thus, it has been shown that Mmp13 deficient mice are more susceptible to bleomycin-induced lung fibrosis while appear to be protected from radiation-induced pulmonary fibrosis [54, 55]. Moreover, no difference in the fibrotic response was observed after hyperoxic lung injury, although Mmp13 deficient mice displayed an increased inflammatory reaction [56]. The reasons for this discrepancy and its possible role of MMP13 in the pathogenesis of IPF are largely unknown.

#### Contribution of the bleomycin model to our understanding of the role of MMPs in lung fibrogenesis

Substantial amount of information regarding the pathogenesis of lung fibrosis come from different animal models where integrative genomic and proteomic have identified several dysregulated MMPs involved in the fibrotic response. Importantly however, bleomycin-induced fibrosis, the most used experimental model, as well as any model of pulmonary fibrosis described so far, does not mimic the pathobiology of IPF, but shares some of the fibrogenic mechanisms thus contributing to our understanding of the human disease.

Following we summarize the findings reported with some other MMPs evaluated in experimental models of lung fibrosis with scarce data in IPF.

#### Mmp12

Mmp12 is one of the most upregulated genes in lungs from bleomycin-treated mice. However, the putative effect on the inflammatory and fibrotic responses has given contradictory results. Thus, while some studies shown that Mmp12-deficiency had no effect on bleomycin-induced inflammation/fibrosis, others reported that MMP12 has a profibrotic role, since Mmp12-null mice have decreased collagen accumulation after several injuries including bleomycin, and anti-Fas antibody [57–59]. Mechanistically, it has been suggested that MMP12 plays an important role in TGF-β1 signaling pathway activation.

#### Mmp14

Several findings indicate that Mmp14 is upregulated in experimental lung fibrosis [36, 37, 60]. MMP14 is a membrane bound enzyme known also known as MT1-MMP that although does not have the classical domain structure of collagenases has been documented as a true collagenase able to degrade collagen fibers [61]. Moreover, it has been shown that MT1-MMP was the sole proteolytic effector of the invasive activity required by human and mice fibroblasts during trafficking through type-I collagen-riche 3-D barriers [62]. We have found that in IPF lungs that MMP14 was the most highly expressed of the membrane type MMPs and was expressed by alveolar epithelial cells, however its role in this disease is presently unknown [63].

#### Mmp28

MMP28 is the last member of the mammalian MMP family and we found that it is upregulated in IPF lungs [7]. However, studies dealing with its possible implication in the pathogenesis of lung fibrosis are scanty. A recent report indicates that Mmp28-null mice are protected from bleomycin-induced lung fibrosis because this deficiency results in higher macrophage influx to the lungs compared with WT mice, and an impaired M1/M2 polarization [64].

### MMPs as prognostic biomarkers in IPF patients

IPF patients display different clinical phenotypes including slowly and rapid progressive clinical courses, and occasional abrupt deterioration due to acute exacerbations. In this context, to predict outcome is very difficult and the development of blood biomarkers may help to identify patients at risk for accelerated disease progression and ideally those who will benefit from therapy.

The search for a peripheral blood protein signature for IPF has suggested that several MMPs could potentially fulfill this role.

In a pioneer study it was revealed that high concentrations of MMP7 and MMP1, together, are sufficient to distinguish IPF patients from patients with COPD, hypersensitivity pneumonitis, sarcoidosis and healthy controls [47]. These results were corroborated in an independent validation cohort including patients with IPF and familial pulmonary fibrosis showing additionally that MMP7 concentrations correlated with the severity of the disease. These findings have been verified by several studies, which demonstrate that levels of circulating MMP7 predict progression and outcome where higher concentrations of this enzyme negatively correlated with pulmonary function tests and survival. More recently, it has been suggested that blood levels of MMP7 may also help to the differential diagnosis between IPF and other fibrotic lung disorders [65, 66]. The mechanisms by which the expression of MMP7 gene and protein increases in IPF is unclear, but may be at least partially related to lower DNA methylation [67]. Also, significant associations between rs11568818AA and rs11568819CT genotypes and elevated plasma levels of MMP7 have been identified. Interestingly, the G-to-A transition of rs11568818 resulted in a novel-binding site for the forkhead box A2 (FOXA2) transcription factor that seems to increase sensitivity of the polymorphic MMP7 promoter to FOXA2 [68]. Therefore, both epigenetic and genetic mechanisms may provide the bases for the upregulation of MMP7 in IPF.

Likewise, the increase of MMP1 may be related to the presence of the 2G/2G genotype at −1,607, which is associated with increased gene expression [69]. Additionally, the sequencing of the MMP1 promoter have revealed a putative gene-environment interaction between the T/G SNP at position −755 and smoking in this disease [69]. This is an important finding because several studies performed in sporadic and familial cases of IPF have shown that smoking is a strong risk factor of IPF [1].

Other MMPs may also be useful to predict outcome but the studies performed so far have been done in small cohorts of IPF patients and without an independent validation. For example, it was observed that an increase in serum MMP3 or MMP10 correlated to disease severity and shortened survival time [70, 71].

Elevated BALF levels of MMP3, 7, 8 and 9 have been reported in rapid compared with slow progressive IPF patients [48]. In a recent study higher concentrations of MMP8 in plasma and BALF levels were corroborated; however, these levels do not correlate with mortality or decline in lung function and it was suggested that this enzyme may not be a prognostic biomarker [50]. Importantly however, most of the IPF patients in the IPF cohort had a history of cigarette smoking while most of the control subjects were non-smokers. Therefore, it cannot be ruled out that higher MMP-8 levels could be associated with the smoking status [50].

Circulating peptides naturally produced by MMPs degradation have been also identified in IPF and proposed as an alternative in the search of informative biomarkers. Using this approach, a recent study demonstrated that changes with time in the concentrations of collagen 1 degraded by MMP2/9/13, collagen 3 degraded by MMP9, collagen 6 degraded by MMP2/9, MMP-degraded biglycan, ADAMTS-degraded collagen 3, and MMP-degraded C-reactive protein, strongly correlate with outcome, both progression and survival [72]. These findings were confirmed in a validation cohort of IPF patients and age-matched and sex-matched healthy controls suggesting that IPF progression and outcome can be determined through detection of temporal change in the concentrations of MMPs degraded circulating proteins having the potential to become theragnostic biomarkers [72].

## Conclusions

In the last two decades, MMPs have emerged as critical players in the pathogenesis of lung fibrosis. However, their participation is not only restricted to their role as ECM modulators, but also determining cell behavior. Crucially, some MMPs seem to promote a fibrotic response while others appear to play a protecting role. In this context, it is important to emphasize that there use as therapeutic targets have to be certain of which specific MMP we want to tackle. Earlier efforts to block MMP activity in cancer patients have failed to achieve clinical success [73]. One of the reasons is that there are structural similarities in the catalytic domain of MMPs and the failed phase III clinical trials have used broad-spectrum small-molecule MMP inhibitors that likely inhibited most of them. Importantly, MMPs may have beneficial and detrimental effects, and in the last years, it has become clear that some individual MMPs such as MMP7 contribute to progression and poor outcome of IPF while others such as MMP19 seems to be protective. Thus, putative inhibitors will need to be highly selective for a particular MMP and able to accumulate in the fibrotic lung without eliciting adverse systemic effects. Moreover, lung remodeling in IPF is a dynamic biopathological process and it is unknown whether the activities of MMPs vary during the course of the disease. Finally, because some functions of MMPs involve non-catalytic domains and even occur in intra-cellular locations, novel protein-binding drugs are needed. Our knowledge of the role of MMPs is still scanty, and further research is needed to develop selective agents as therapeutic targets
